# Flame Imaging Technology Based on 64-Pixel Area Array Sensor

**DOI:** 10.3390/mi15010044

**Published:** 2023-12-25

**Authors:** Xiaodong Huang, Xiaojian Hao, Baowu Pan, Xiaodong Liang, Zheng Wang, Shenxiang Feng, Pan Pei, Heng Zhang

**Affiliations:** 1 Science and Technology on Electronic Test and Measurement Laboratory, North University of China, Taiyuan 030051, China; b200608@st.nuc.edu.cn (X.H.); s202206106@st.nuc.edu.cn (X.L.); sz202206084@st.nuc.edu.cn (Z.W.); sz202106015@st.nuc.edu.cn (S.F.); b20210622@st.nuc.edu.cn (P.P.); 2 School of Materials Science and Engineering, North University of China, Taiyuan 030051, China; pan_mail@nuc.edu.cn; 3 Shanxi Key Laboratory of Advanced Semiconductor Optoelectronic Devices and System Integration, Jincheng 048000, China; zhangheng@jcgjd.org.cn

**Keywords:** TDLAS, combustion flame, ART, two-dimensional imaging

## Abstract

High-resolution flame temperature images are essential indicators for evaluating combustion conditions. Tunable diode laser absorption spectroscopy (TDLAS) is an effective combustion diagnostic method. In actual engineering, due to the limitation of line-of-sight (LOS) measurement, TDLAS technology has the problems of small data volume and low dimensionality in measuring combustion fields, which seriously limits the development of TDLAS in combustion diagnosis. This article demonstrates a TDLAS imaging method based on a 64-pixel area array sensor to reconstruct the two-dimensional temperature field of the flame. This paper verifies the robustness of the Algebraic Reconstruction Technique (ART) algorithm through numerical simulation and studies the effects of temperature, concentration, and pressure on the second harmonic intensity based on the HITRAN database. The two-dimensional temperature field of the flame was reconstructed, and reconstruction accuracy was verified using thermocouples. The maximum relative error was 3.71%. The TDLAS detection system based on a 64-pixel area array sensor provides a way to develop high-precision, high-complexity flame temperature measurement technology.

## 1. Introduction

The proper distribution of the temperature field and concentration field in the combustion environment is a powerful tool for combustion diagnosis, reflecting the uniformity and evolution of combustion. The instability of the combustion field has always been a long-standing problem in the design of combustion chambers in aircraft engines, gas turbines, etc. The reason is the lack of high dynamic thermodynamic parameters [1,2,3]. The distribution of high-resolution physical fields will help researchers analyze combustion conditions more accurately [4]. Therefore, accurate measurement of combustion fields is essential in solving the temperature field distribution and evaluating combustion in complex combustion environments such as high-temperature, high-pressure, and high impact environments.

At present, the testing and performance evaluation of high-energy combustion temperature fields represented by the exit of the aero-engine combustion chamber is mainly divided into two methods: contact temperature measurement and non-contact temperature measurement [5,6,7,8,9]. The former temperature measurement principle is simple. The sensing device only needs to be placed in the area to be measured. When the ambient temperature changes, it can quickly respond to the temperature change, convert the detection signal into an electrical signal, and transmit it to the data acquisition module of the host computer. Calibration establishes the corresponding relationship between electrical signals and temperature information to collect temperature signals. However, it is easily damaged in highly complex environments; the non-contact temperature measurement method relies on detecting other easily measurable elements in the field caused by temperature changes. Characteristic changes in physical parameters use the physical parameters as carriers to represent temperature information outwards. Commonly used non-contact temperature measurement methods mainly include the radiation temperature measurement method [10,11,12], the absorption spectrum temperature measurement method [13,14,15,16], and the acoustic wave temperature measurement method [17,18].

Tunable semiconductor laser absorption spectroscopy (TDLAS) [19] is a technology that can measure multiple parameters, such as temperature, substance concentration, and the pressure of methane, oxygen, carbon dioxide, water vapor, etc. It can also be based on the measurement results of a single gas. Derivatization measures component concentrations of multiple substances. Based on the measured absorption peak waveform under different environmental conditions (such as measuring optical path, measuring environmental pressure, measuring environmental temperature, etc.) and controlling multiple environmental condition variables, another environmental condition value can be obtained, such as controlling optical path and pressure parameters and solving for temperature, etc. Tunable diode laser absorption spectroscopy (TDLAS) has been successfully used in the monitoring of various industrial occasions such as thermal power plants, aerospace engines, and wind tunnels due to its high accuracy, fast response speed, low cost, and stable performance [20,21,22].

Kamimoto T [23,24] et al. reported a dynamic combustion flow research technology based on broadband and frequency layer analysis absorption spectroscopy, applied 16-channel CT-TDLAS to Bunsen burners, and obtained the CH_4_ concentration and two-dimensional temperature distribution. The research team of the Hefei Institute of Physical Sciences, Chinese Academy of Sciences, and Xia [25,26] selected the absorption spectral lines of water molecules for measurement, discussed the impact of different projection angles and the number of parallel rays on the reconstruction error, and proved that the reconstruction error varies with the projection angle and parallel rays. It increases with the decrease in the number of rays, and reconstruction accuracy depends more on the number of parallel rays than on the number of projection angles. Xu’s team [27,28,29] built a TDLAS detection system using a five-viewpoint fan-shaped laser beam sensing array based on the distribution of H_2_O in the combustion environment. They optimized the TDLAS wavelength modulation spectrum detection method by combining frequency division multiplexing and prominent peak scanning technology, improving the TDLAS detection frame rate. The temperature and H_2_O concentration were reconstructed using a five-view fan beam detection tomography system.

This paper proposes a TDLAS technology based on a 64-pixel area array sensor to achieve high-resolution temperature field measurement. First, numerical simulation was used to verify the accuracy of the ART algorithm, with a relative error of ±6%. The effects of temperature, concentration, and pressure on the second harmonic intensity were studied through the HITRAN database [30], and the two-dimensional butane flame was then studied. The temperature field was imaged and reconstruction accuracy was finally verified by taking temperature measurements using thermocouples. The maximum relative error was 3.71%. It provides a way to develop high-precision flame temperature imaging technology.

## 2. Materials and Methods

### 2.1. Tunable Diode Laser Absorption Spectroscopy

The mathematical principle of TDLAS is the Beer–Lambert law [31]. Its physical significance is that when a beam of incident light with intensity *I*_0_ passes through the area to be measured, a transition resonance occurs with the target molecule, the energy of the incident light is absorbed, and the attenuated outgoing light intensity *I_t_* is received by the optical sensor. The functional expression of the transmission coefficient *τ*(*ν*) of laser center frequency *ν* (cm^−1^) can be expressed as follows:

(1) $$\tau (\nu )=\frac{I_{t}}{I_{0}}=\exp\left\{-\int_{0}^{L}P(x)\cdot X(x)\cdot S[T(x)]\cdot \phi (\nu )dx\right\}$$

where *L* (cm) is the absorption optical path length, *P* (atm) is the environmental pressure of the area to be measured, *X* is the molar concentration of the absorbing component, *S*(*T*) is the intensity of the spectral line, *T* (K) is the temperature of the gas to be measured, and the absorption spectrum line function *ϕ* (*ν*) satisfies normalization.

In the WMS method [32], a high-frequency modulation signal with frequency *f_m_* drives the DFB laser to produce a spectrum scan, and the incident laser intensity can be expressed as

(2) $$I_{i}(t)={\bar{I}}_{i}[1+a\cos(2\pi f_{m}t)]$$

where ${\bar{I}}_{i}$ and *a* are the unmodulated incident laser intensity and the modulated laser intensity coefficient, respectively. The laser wavelength also changes periodically with the same frequency, i.e.,

(3) $$\nu (t)=\bar{\nu }(t)+b\cos(2\pi f_{m}t+\varphi )]$$

In the formula, $\bar{\nu }(t)$ is the slow scanning signal, *b* is the modulation amplitude, *f_m_* is the modulation frequency, and *φ* is the phase difference between the wavelength and intensity profile in the modulated laser. The first harmonic and second harmonic of the transmitted laser intensity are expressed as *H*_1*f*_ and *H*_2*f*_, respectively. When the absorption is weak, at the central wavelength ${\bar{\nu }}_{0}$ of the absorption spectrum, the normalized second subharmonic *H*_2*f*/1*f*_ can be expressed as

(4) $$H_{2f/1f}=\frac{H_{2f}}{H_{1f}}\approx \frac{P}{a}\int_{0}^{l}X(x)S[T(x)]\phi (\bar{\nu },b)dx$$

(5) $$\phi (\bar{\nu },b)=-\frac{1}{\pi }\int_{-\pi }^{+\pi }\phi (\bar{\nu }+b\cdot \cos\theta )\cos(2\theta )d\theta$$

**Figure 1:**
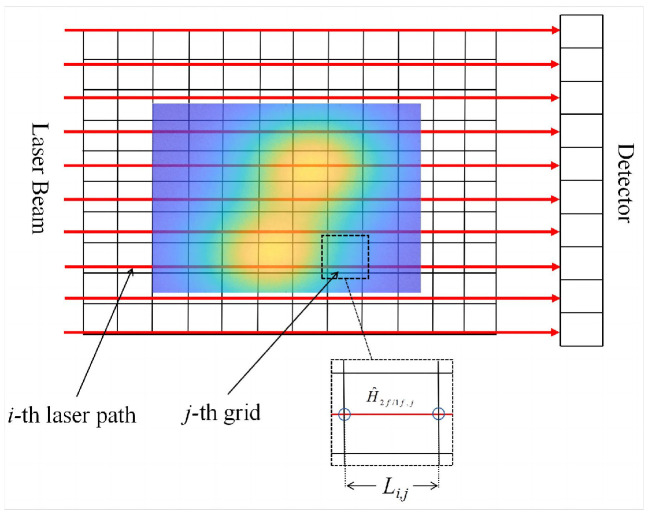
Schematic diagram of parallel laser projection and ROI discretization structure.

The results show that the normalized second subharmonic *H*_2*f*/1*f*_ is a parameter integrated along the laser path, and the tomographic method can recover the two-dimensional distribution of absorption parameters such as temperature and substance concentration. In tomography, the laser intensity of multiple angle laser paths is used to obtain the normalized second harmonic of the area of interest from the WMS method and tomographic images, and the temperature distribution of the combustion field is reconstructed through an algorithm. Figure 1 depicts a square region of interest that is uniformly discretized into N grids. In each grid, parameters such as combustion field temperature, pressure, and gas concentration are assumed to be constant.

The absorption path length of the *i*-th laser path in the *j*-th grid can be recorded as *L_i_*_,*j*_. Therefore, the *Ĥ* _2*f*/1*f*_ value of the center wavelength of the absorption spectrum *ν*_0_ obtained by the *i*-th projection can be expressed by the laser in the *j*-th grid. The local normalized second harmonic *Ĥ*_2*f*/1*f*,*j*_ with center frequency *ν* is expressed as

(6) $$H_{2f/1f,i}=\sum_{j}^{N}l_{i,j}\cdot {\hat{H}}_{2f/1f,j}$$

To simplify the calculation, Equation (7) and the matrix represented by Equation (8) can usually be substituted, where *L* is the absorption path length.

(7) $$\left\{\begin{array}{l} H_{2f/1f,1}={\hat{H}}_{2f/1f,1}L_{1,1}+{\hat{H}}_{2f/1f,2}L_{1,2}+\cdots {\hat{H}}_{2f/1f,j}L_{1,j}\\ H_{2f/1f,2}={\hat{H}}_{2f/1f,1}L_{2,1}+{\hat{H}}_{2f/1f,2}L_{2,2}+\cdots {\hat{H}}_{2f/1f,j}L_{2,j}\\ \cdots \\ \cdots \\ H_{2f/1f,i}={\hat{H}}_{2f/1f,1}L_{i,1}+{\hat{H}}_{2f/1f,2}L_{i,2}+\cdots {\hat{H}}_{2f/1f,j}L_{i,j} \end{array}\right.$$

(8) $$H_{2f/1f}=L\cdot {\hat{H}}_{2f/1f}$$

### 2.2. ART Theory

The ART algorithm solves the system of equations on the projection data by establishing reference matrix coefficients [33,34,35]. Iterative calculations are performed starting from the initial values to find the best solution to the system of equations. ART is a very effective method when the projection data corresponding to the projected sinogram are insufficient or not uniformly distributed.

The iterative reconstruction problem can be reduced to finding the following system of linear equations:

(9) $$\left\{\begin{array}{l} \begin{array}{c} a_{11}*f_{1}+a_{12}*f_{2}+\cdots +a_{1n}*f_{n}=d_{1}\\ a_{21}*f_{1}+a_{22}*f_{2}+\cdots +a_{2n}*f_{n}=d_{2}\\ \vdots \end{array}\\ a_{m1}*f_{1}+a_{m2}*f_{2}+\cdots +a_{mn}*f_{n}=d_{m} \end{array}\right.$$

Here, Equation (10) is called the projection matrix coefficient, which is determined by the system, including the emitter’s ray angle, the object’s position, the detector resolution, etc.

(10) $$\left[\begin{array}{cccc} a_{11} & a_{12} & \cdots & a_{1n}\\ a_{21} & a_{22} & \cdots & a_{2n}\\ \vdots & \vdots & \ddots & \vdots \\ a_{m1} & a_{m2} & \cdots & a_{mn} \end{array}\right]$$

The essence of the ART algorithm is to solve the problem of linear equations, and it is a fundamental reconstruction algorithm. When the number of unknowns in the system of equations is greater than the number of equations, it is necessary to search for appropriate solutions by adding a priori information and restrictive conditions. The implementation process of the ART algorithm is roughly divided into six stages:

(1) Discretely divide the area to be reconstructed into a grid with a specific resolution. Usually, the resolution of this grid is higher than the resolution of the detector;
(2) Combine the projection equations corresponding to the projection rays for each pixel of the detector to form a linear equation system, where each equation contains the object attenuation coefficient;
(3) Assign an initial value to the projection weight of the discrete grid obtained in step (1), and calculate the projection coefficient of each projection ray based on this;
(4) Starting from the first projection equation, solve the calculated projection value and obtain the difference with the real projection value. According to the iterative formula, gradually correct the discrete grid projection weight;
(5) Use the corrected projection weight value to be brought into the second projection equation, and perform similar operations in (4) until the last ray to complete an iteration;
(6) Repeat steps (4) and (5), except that the latest projection weight coefficient is used starting from the first equation for the second iteration until, after several iterations, the two adjacent grid projections are the difference in weight values, which will already be very small. The calculation ends, and the solution to the linear equations is finally obtained.

## 3. Simulation Analysis

### 3.1. Numerical Simulation

**Figure 2:**
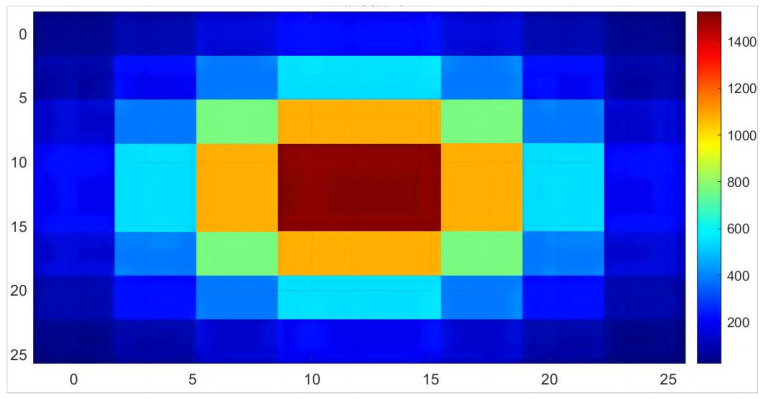
Raw numerical simulation image.

According to the above ART principle, we reconstructed and simulated the waveform of unimodal Gaussian distribution. In this article, the original image with 8 × 8 resolution was simulated, as shown in Figure 2, and was projected and reconstructed. The main factors affecting the reconstruction speed include projection coefficient solution, iteration order, relaxation factor selection, and prior conditions. Among them, the projection coefficient is the key to affecting the iteration speed, and its solution method is also the one with the most optimization potential among the above factors. Based on the principle of laser gas absorption and detector characteristics, the projection matrix coefficient is determined based on the plane area each beam passes through and the relationship between this area and the sensor pixel position.

**Figure 3:**
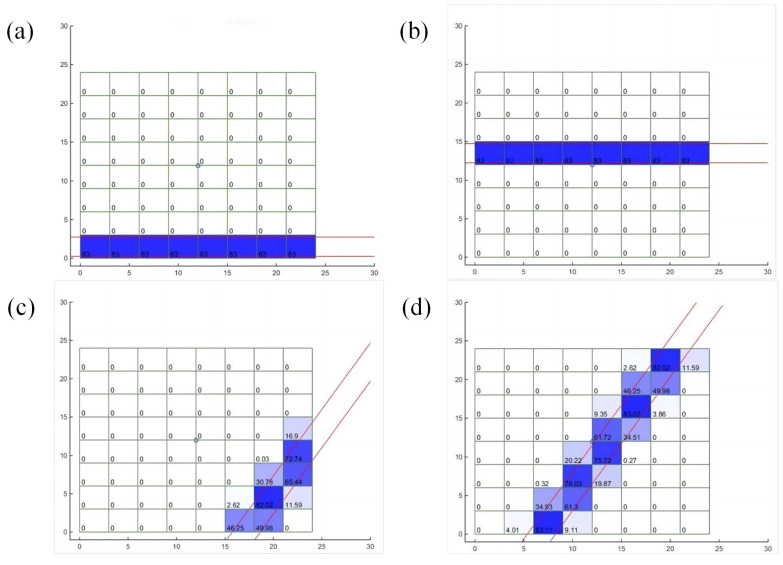
Weight of sensor pixels: (**a**) represents the light diagram of the first pixel when the detection angle is 0°; (**b**) represents the light diagram of the fifth pixel when the detection angle is 0°; (**c**) represents the light diagram of the first pixel when the detection angle is 60°; and (**d**) represents the light diagram of the fourth pixel when the detection angle is 60°.

According to the relationship between different incident angles and different sensor pixels, the transmission path of the corresponding light can be simulated, as well as the weight proportion coefficient in each divided grid area, as shown in Figure 3 below (Figure 3a and b are detections in light diagrams when the angle is 0° for the first and fifth pixels, respectively). Figure 3c,d are the light diagrams of the first and fourth pixels when the detection angle is 60°. They will be measured or simulated. After all the weight-proportional coefficients corresponding to the angles are calculated, the initial coefficients can be obtained in order to solve the system of equations.

**Figure 4:**
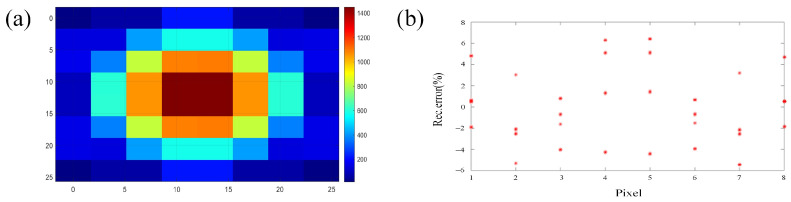
Reconstruction results: (**a**) two-dimensional reconstructed image; (**b**) relative error map.

The weight coefficient matrix obtained by the above method is combined with the sinogram data obtained by projecting the original graphics to perform ART calculation. The reconstruction result is shown in Figure 4a,b is the relative error diagram of the reconstruction result.

It can be seen that the relative error of ART’s reconstruction of the 8 × 8 unimodal Gaussian waveform is ±6%. At the signal intensity distribution of 64 pixels, the reconstructed waveform is basically consistent with the original waveform.

### 3.2. Analysis of the Influence of Physical Quantities

In TDLAS technology, different temperatures, pressures, and concentrations will cause changes in spectral lines, resulting in significant errors. Based on the HITRAN database, this article analyzes the waveforms of the measurement object (O_2_) with center frequencies of 13,142.54 cm^−1^ and 13,154.18 cm^−1^ in subsequent experiments. Simulation analysis under different parameters was conducted to help the accuracy of subsequent experimental measurements. We set the initial temperature to 296 K, the concentration to 21%, and the pressure to 1 atm.

**Figure 5:**
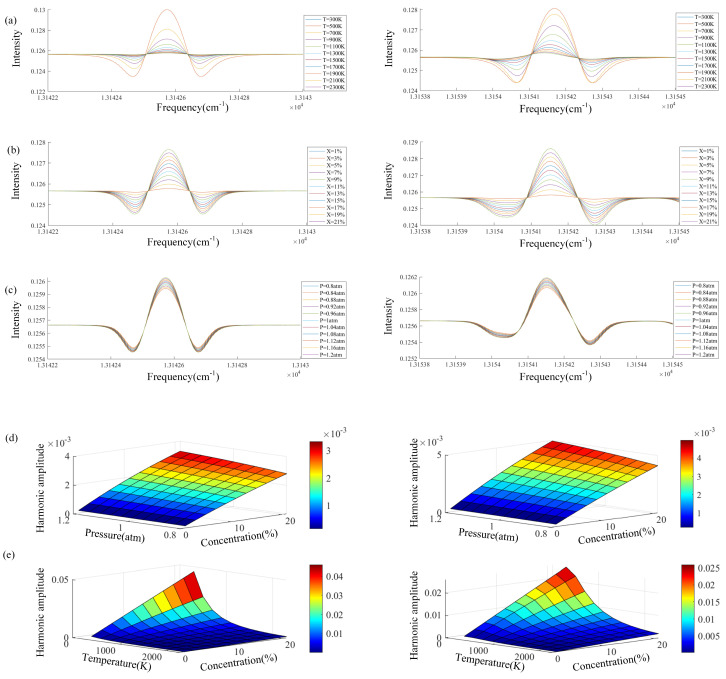
Relationship between temperature, concentration, pressure, and the amplitude of two wavelengths: (**a**) the effect of different temperatures on the amplitude of two wavelengths; (**b**) the effect of different concentrations on the amplitude of two wavelengths; (**c**) the effect of different pressure on the amplitude of two wavelengths; (**d**) effect of different concentration and pressure on the amplitude of two wavelengths; (**e**) effect of different concentration and temperature on the amplitude of two wavelengths.

When studying the effect of temperature on waveforms, concentration and pressure are kept constant to control the effects of other variables on spectral lines. The effect of temperature on the second harmonic is shown in Figure 5a. Lines of different colors represent the intensity of the second harmonic under the influence of temperature. It can be seen that the intensity is inversely proportional to temperature. When studying the effect of concentration on waveform, the temperature and pressure remain unchanged, and the effect of concentration on intensity is shown in Figure 5b. It can be found that as the concentration increases, the intensity also increases. The Doppler line width and the collision line width determine the spectral line width. The Doppler line width is only related to the wave number and temperature and does not change much. The collision line width changes significantly when it is affected by pressure. However, when the pressure change is small, the line width does not change significantly, and the pressure only affects the intensity. When studying the influence of pressure on the waveform, the temperature and concentration remain unchanged. The influence of pressure on intensity is shown in Figure 5c. As the pressure increases, the strength also increases. Figure 5d and Figure 5e respectively show the influence of the other two variables on the second harmonic intensity when the temperature remains unchanged at 1500 K and the pressure remains unchanged at 1 atm. It can be seen that when the other two conditions change, the change in the second harmonic intensity is negatively correlated with temperature, positively correlated with concentration, and positively correlated with pressure. In the experiment, a high-concentration gas and a high-pressure combustion environment were selected to measure the temperature field.

## 4. Experiment

**Figure 6:**
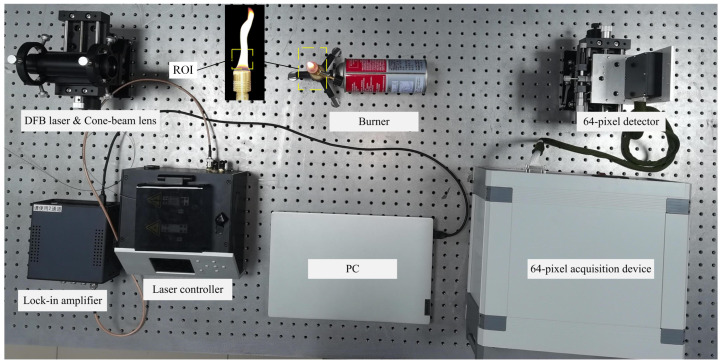
Experimental device diagram.

This paper selects a relatively stable butane flame as the reconstruction object. The experimental device is shown in Figure 6. The laser driver (Microphotons, Shanghai, China) generated a triangular superimposed sinusoidal wave signal through the signal generator (DG5252, Suzhou, China). The laser was cylindrically parallel to the beam expander lens. Light: the beam diameter was 30 mm, the flow field to be measured was a Bunsen burner flame, the flow rate was 3 mL/min, the flame height was 60 mm, and the diameter was 15 mm. After non-premixed combustion, the detector (Hamamatsu S13620-02, Shizuoka, Japan) received and collected the signal. Projection data were collected at 0°, 60°, 120°, and 180°. The flame temperature distribution was subsequently reconstructed.

**Figure 7:**
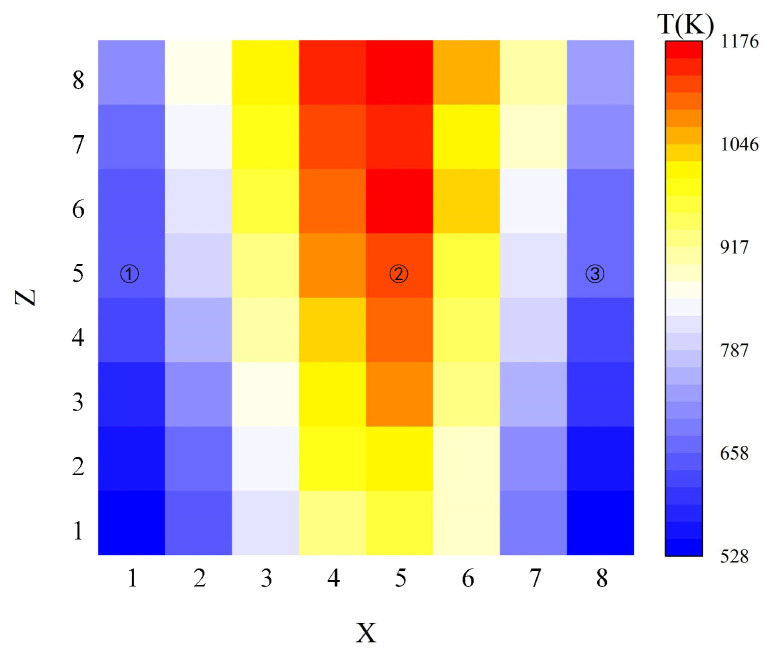
Flame reconstruction temperature map.

This article sets the region of interest (ROI) as a square area with a length of 2.5 cm, uses the central section as the two-dimensional temperature field to reconstruct the flame image, verifies the reconstruction effect of the algorithm on the flame temperature field, and collects three measurement values in the ROI through thermocouples for comparison with the reconstructed temperature field to finally determine the accuracy of the temperature field reconstruction. The reconstruction results of the two-dimensional temperature field of the flame are shown in Figure 7. The temperature of the two-dimensional temperature field is the largest at the center position, and the temperature gradually decreases from the center position to the edge position. As the flame height increases, the temperature field gradually increases, consistent with flame temperature changes.

This article uses K-type thermocouples to measure the temperature values at three sampling points at the center and the left and right edges of Figure 7 and calculate the relative error. The results are shown in Table 1. Comparing the reconstruction results with the thermocouple measurement values, their minimum relative error is 3.01% and the maximum is 3.71%. The ART algorithm has the best reconstruction effect at sampling point 2. Decreases in thermocouple measurements may be due to heat transfer.

## 5. Conclusions

In summary, we report a method for flame temperature imaging based on TDLAS with a 64-pixel area array sensor. This article improves the spatial resolution of the test system and solves the problem of small data volume. The improved ART algorithm provides higher accuracy and faster reconstruction speed in multi-dimensional temperature field reconstruction. Numerical simulation shows that the ART algorithm has good robustness. Based on the HITRAN database, the effects of temperature, concentration, and pressure on the second harmonic intensity were simulated, and it was found that the second harmonic intensity was negatively correlated with temperature, positively correlated with concentration, and positively correlated with pressure. In the combustion field reconstruction experiment, thermocouple points were used for verification, and the relative error was within 4%, indicating that the ART algorithm can express the actual combustion temperature field. The TDLAS system based on the 64-pixel area array sensor improves its value in engineering applications and provides a means of high-resolution measurement imaging in TDLAS.

## Figures and Tables

**Table 1:** Error analysis based on thermocouples.

Position	Thermocouple Measurements (K)	Reconstructed Value (K)	Relative Error (%)
1	619	642	3.71
2	1095	1128	3.01
3	637	658	3.29

## Data Availability

Data are contained within the article.
